# Rapid functional analysis of computationally complex rare human *IRF6* gene variants using a novel zebrafish model

**DOI:** 10.1371/journal.pgen.1007009

**Published:** 2017-09-25

**Authors:** Edward B. Li, Dawn Truong, Shawn A. Hallett, Kusumika Mukherjee, Brian C. Schutte, Eric C. Liao

**Affiliations:** 1 Center for Regenerative Medicine, Massachusetts General Hospital, Harvard Medical School, Boston, Massachusetts, United States of America; 2 Harvard Stem Cell Institute, Harvard University, Cambridge, Massachusetts, United States of America; 3 Departments of Microbiology and Molecular Genetics, and Pediatrics and Human Development, Michigan State University, East Lansing, Michigan, United States of America; Mayo Clinic College of Medicine, UNITED STATES

## Abstract

Large-scale sequencing efforts have captured a rapidly growing catalogue of genetic variations. However, the accurate establishment of gene variant pathogenicity remains a central challenge in translating personal genomics information to clinical decisions. Interferon Regulatory Factor 6 (*IRF6*) gene variants are significant genetic contributors to orofacial clefts. Although approximately three hundred *IRF6* gene variants have been documented, their effects on protein functions remain difficult to interpret. Here, we demonstrate the protein functions of human *IRF6* missense gene variants could be rapidly assessed in detail by their abilities to rescue the *irf6* ^*-/-*^ phenotype in zebrafish through variant mRNA microinjections at the one-cell stage. The results revealed many missense variants previously predicted by traditional statistical and computational tools to be loss-of-function and pathogenic retained partial or full protein function and rescued the zebrafish *irf6* ^*-/-*^ periderm rupture phenotype. Through mRNA dosage titration and analysis of the Exome Aggregation Consortium (ExAC) database, *IRF6* missense variants were grouped by their abilities to rescue at various dosages into three functional categories: wild type function, reduced function, and complete loss-of-function. This sensitive and specific biological assay was able to address the nuanced functional significances of *IRF6* missense gene variants and overcome many limitations faced by current statistical and computational tools in assigning variant protein function and pathogenicity. Furthermore, it unlocked the possibility for characterizing yet undiscovered human *IRF6* missense gene variants from orofacial cleft patients, and illustrated a generalizable functional genomics paradigm in personalized medicine.

## Author summary

Advances in sequencing technologies have led to rapid increases in personalized genetics information. Millions of differences exist when comparing the genomes of two individuals, accounting for the diversity of humans and occasionally disease pathogenicity. Accurate determination of the functional consequences of these differences is critical for translating personal genetic information into clinical decision. Various methods have been devised to meet this challenge. However, they are imperfect, especially in their abilities to interpret rare variants. Rare variants must be evaluated by multi-pronged approaches to establish disease pathogenicity, one crucial approach being functional testing through experimental models. Here, we utilized a zebrafish model to rapidly evaluate the protein functions of rare gene variants of *IRF6*, a gene of established importance in orofacial cleft pathogenesis. *IRF6* functions are well-conserved across species, and allowed us to test the functions of human *IRF6* variants by their abilities to rescue zebrafish embryos depleted of *irf6*. Many variants previously labeled pathogenic and loss-of-function retained full wild type-level protein activity, suggesting they could potentially represent benign, rather than disease-causing genetic variations. This paradigm is applicable to other genes and will allow researchers to rapidly triage gene variants for further study and clinicians to provide more accurate genetic diagnoses.

## Introduction

The rapid development of next-generation sequencing technologies has ushered in a new era of personalized medicine for a myriad of diseases [1]. Large-scale consortia sequencing efforts have documented thousands of whole exomes and genomes from both disease patients and the general population, and captured a growing catalogue of genetic variations for statistical comparisons and analyses [2]. However, a frequent challenge in the analysis of human gene variants is the establishment of pathogenicity for disease, distinguishing disease-causing variants from the background of variants present across human populations that are rare and undetermined in function, but not actually pathogenic. Statistical methods based on the relative enrichment of certain gene variants in disease populations [3–6], and computational methods based on sequence conservation or structural information with limited biological data are often inadequate and provide conflicting results [7,8]. Indeed, false assignment of pathogenicity for gene variants is a critical challenge in translating knowledge gained from genome sequencing to clinical diagnoses and treatments. One focused resequencing study recently revealed that as many as 27% of previously published disease-causing variants were benign or lacked sufficient evidence for pathogenicity and therefore should be categorized as variants of unknown significance [9]. In addition, the Exome Aggregation Consortium (ExAC) recently published a study utilizing the largest aggregation of human exomes to reveal that while each person has an average of 54 variants in their genome that are currently annotated as pathogenic, as many as 41 of them are now observed to occur frequently in the human population and thus are unlikely to cause disease [10]. As the amount of exome/genome sequencing data continues to increase exponentially, it is crucial for candidate gene variants to undergo rigorous, multi-pronged evaluation before pathogenicity assignments. In addition to statistical (case-control association, familial segregation, population frequency, and etc.) and bioinformatic (evolutionary conservation, protein energetics, and etc.) methods, experimental approaches utilizing biological assays that directly test the protein functions of gene variants should be implemented to provide functional evidence that directly links gene variants to the pathogenesis of disease [11].

One well-studied example of a common congenital malformation associated with gene variants is that of orofacial clefts associated with the transcription factor Interferon Regulatory Factor 6 (*IRF6*, ENSG00000117595) [12–15]. Orofacial clefts are among the most common congenital malformations with an estimated incidence of approximately 1 in 700 births [16]. Pathogenic variants in *IRF6* are among the most common genetic causes of cleft lip and/or palate (CL/P) [17] and are associated with both Van der Woude syndrome (VWS, OMIM 119300) and Popliteal Pterygium syndrome (PPS, OMIM 119500), autosomal dominant Mendelian disorders with variable penetrance and expressivity that are characterized by CL/P and skin abnormalities [12]. The *IRF6* gene sequence is highly conserved across vertebrates and contains two functional domains, a helix-turn-helix DNA-binding domain and a SMIR/IAD protein-binding domain [12,18]. From murine studies, disruption of *Irf6* led to a CL/P phenotype in addition to oral epithelial adhesions, poor epithelial barrier functions, and improper skin stratification, revealing a potentially important role for the oral epithelium in directing palate development [19,20]. Approximately 300 human *IRF6* gene variants have been catalogued [15], and despite the wealth of structural and biological data for this well-described transcription factor, accurate determinations of variant protein functions and pathogenicity assignments associated with *IRF6* gene variants remain significant challenges. Moreover, different computational programs use algorithms that weight various aspects of amino acid change differently and often provide conflicting predictions on protein function for the same missense mutation [21].

The large number of human *IRF6* gene variants and its well-understood biology make *IRF6* an ideal model to examine the challenges of assigning variant protein function and pathogenicity. In order to develop a rapid functional assay to determine the protein functions of a large number of *IRF6* gene variants, we utilized a novel *irf6* null zebrafish model by taking advantage of the finding that maternally-deposited *irf6* transcripts are required for the proper development of the embryonic epithelium (periderm) during epiboly [22]. By using CRISPR-Cas9 to generate a zebrafish *irf6* null model, we were able to establish maternal-null *irf6* ^*-/-*^ homozygotes where 100% of the embryos lacked Irf6 and ruptured unless rescued by functional Irf6 protein. This *irf6* rescue assay was used to test the protein functions of human *IRF6* missense gene variants, and provide an additional line of biological evidence to help bridge the gap between gene variant identification and pathogenicity assignment.

## Results

### CRISPR disruption of *irf6* in zebrafish reveals crucial role of *irf6* maternal transcripts during epiboly

**Fig 1:**
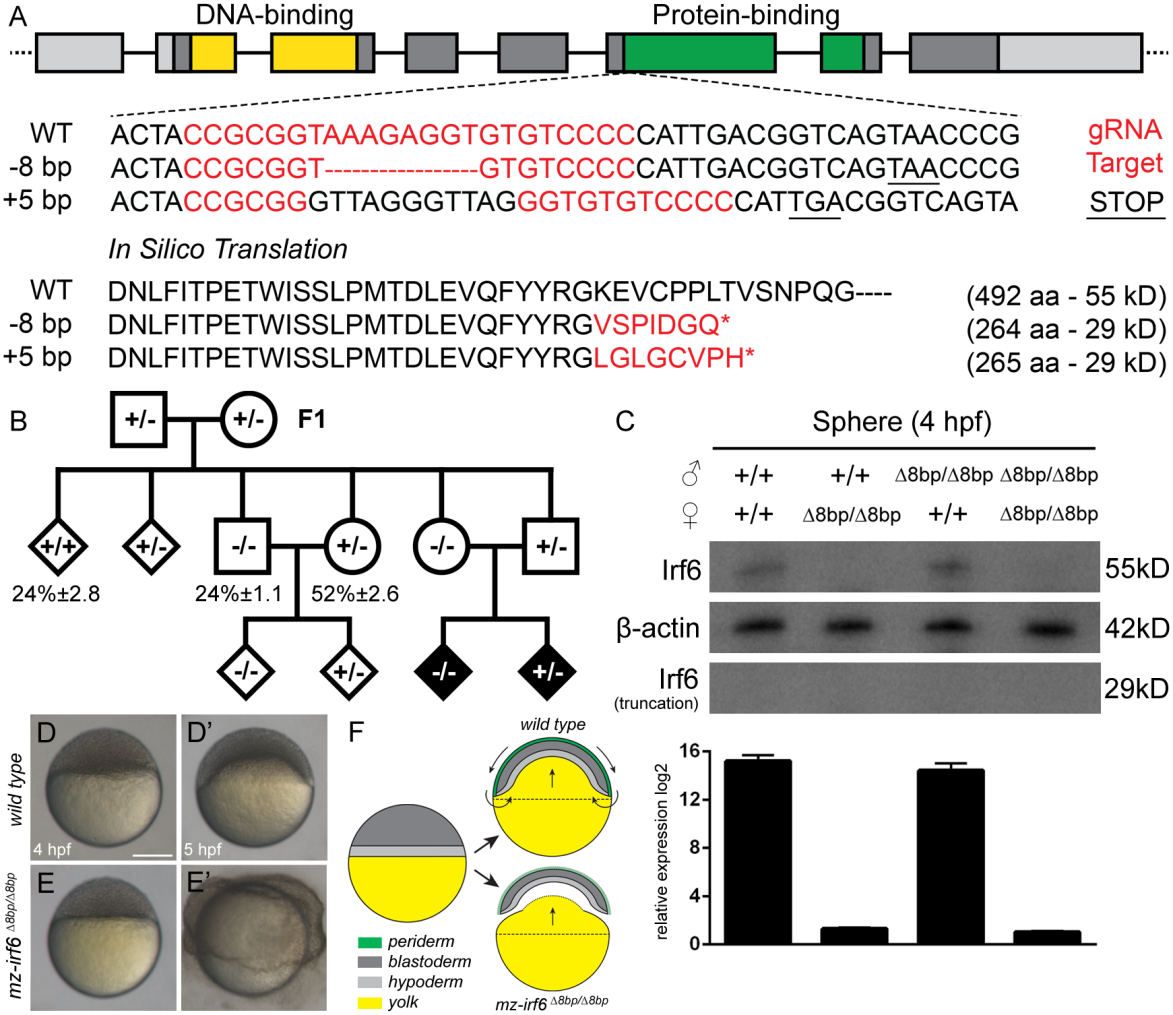
Generation and characterization of the zebrafish *irf6* CRISPR mutant model. **(A)** Zebrafish *irf6* gene structure composed of eight exons, a helix-turn-helix DNA-binding domain (yellow), and a SMIR/IAD protein-binding domain (green). The CRISPR gRNA target site was located in exon 6 at the start of the protein-binding domain. Sanger sequencing of the target site revealed a -8bp deletion (Δ8bp) that created a frameshift and premature stop codon, truncating the protein to 29 kD as predicted by in silico translation. Another +5bp insertion was also identified. **(B)** Breeding pedigree revealed the *irf6* mutant phenotype in F3 and the importance of maternal transcripts. **(C)** Top: western blot at the sphere stage (4 hpf) revealed a lack of Irf6 full-length (55 kD) or truncated (29 kD) protein in all maternal *irf6* ^*Δ8bp/Δ8bp*^ embryos but not paternal *irf6* ^*Δ8bp/Δ8bp*^ embryos or wild type embryos. Bottom: relative gene expression by RT-qPCR revealed a lack of *irf6* mRNA transcripts in all maternal *irf6* ^*Δ8bp/Δ8bp*^ embryos but comparable levels between wild type and paternal *irf6* ^*Δ8bp/Δ8bp*^ embryos. Error bar = 2xSEM, n = 3. **(D-D’)** Wild type embryos at the sphere stage (4 hpf) (D) and at the 30% epiboly stage (5 hpf) (D’). **(E-E’)** Maternal *irf6* ^*Δ8bp/Δ8bp*^ embryos at the sphere stage (4 hpf) (E) and displaying the periderm rupture phenotype at 5 hpf (E’). Scale bar = 250 μm. **(F)** Cross-sectional schematic through the embryonic midline illustrating the zebrafish embryo epiboly process. Arrows represent cell and yolk directional movements. Wild type embryos experience rapid cellular lamination and yolk doming between 4–5 hpf, while maternal-zygotic *irf6* ^*Δ8bp/Δ8bp*^ embryos experience incomplete periderm differentiation and animal pole/yolk separation.

To establish a zebrafish *irf6* null model and investigate the role of *irf6* in periderm and craniofacial development, CRISPR-Cas9 was used for mutagenesis targeted at exon 6 of *irf6* at the start of the SMIR/IAD protein-binding domain (Fig 1A). CRISPR-injected P0 embryos were raised to adulthood, out-crossed with wild type Tü adults, and genotyped at the gRNA target site. A F1 line was identified containing an 8 bp deletion in exon 6 of the *irf6* coding region (NC_007133.7(NM_200598.2):c.772_779del), here forward referred to as “Δ8bp” (Fig 1A). Heterozygous F1 embryos (*irf6* ^*+/Δ8bp*^*)* were in-crossed to produce wild type, heterozygous and homozygous progeny at the expected Mendelian ratios with normal embryonic and craniofacial development (Fig 1B).

It has been previously reported through gene expression analysis and experiments involving dominant-negative Irf6 that maternal transcripts of *irf6*, deposited in the oocyte cytoplasm during gametogenesis, were critical for proper periderm differentiation and epiboly progression [22,23]. The zebrafish periderm is an embryonic epithelium with many morphologic and molecular features similar to the mammalian embryonic oral epithelium that surrounds the facial prominences [24,25]. Based on these previous reports of *irf6* maternal contributions, homozygous *irf6* ^*Δ8bp/Δ8bp*^ F2 females were crossed with males of any genotype to produce heterozygous (*+/Δ8bp*) and homozygous (*Δ8bp/Δ8bp*) *irf6* embryos that developed normally up to the sphere stage (4 hours post fertilization = hpf). However, shortly thereafter these maternal *irf6* ^*Δ8bp/Δ8bp*^ embryos, regardless of genotype, failed to appropriately initiate epiboly compared to wild type embryos, resulting in the separation of the animal pole structures from the underlying yolk, periderm rupture, and embryonic lethality in 100% of embryos between 5–6 hpf (Fig 1D, 1D’, 1E and 1E’). Conversely, when homozygous F2 males were crossed with wild type or heterozygous females, the resulting embryos underwent normal epiboly progression and embryonic development, confirming that maternal contributions of *irf6*, even from heterozygous *irf6* ^*+/Δ8bp*^ females, were sufficient for epiboly progression (Fig 1B). Another zebrafish *irf6* line harboring a 5 bp insertion in exon 6 of the *irf6* coding region was also identified from the same CRISPR-injected P0 embryos and produced the same set of phenotypes as the Δ8bp deletion line (Fig 1A).

### Indel mutations in *irf6* result in mRNA instability and significantly reduced protein expression

The 8bp deletion in the *irf6* coding region was predicted by *in silico* translation to cause a frameshift and nonsense mutation downstream of the CRISPR gRNA target site, truncating the resulting protein to 264 amino acids (29 kD) compared to the complete wild type 492 amino acids (55 kD) (Fig 1A). It has been previously reported that some missense and nonsense mutations in exon 6 of *irf6* resulted in Irf6 proteins with dominant-negative activities [12]. Thus, in order to characterize the *irf6* Δ8bp zebrafish model at the molecular level, RT-qPCR using primers specific to the *irf6* 5’ UTR overlapping the protein N-terminus was performed on zebrafish embryos at the sphere stage (4 hpf) from permutations of wild type and maternal/paternal homozygous *irf6* ^*Δ8bp/Δ8bp*^ crosses. Transcript levels of *irf6* were undetectable in all maternal *irf6* ^*Δ8bp/Δ8bp*^ crosses at 4 hpf, regardless of whether the embryos were homozygous or heterozygous for the Δ8bp *irf6* allele (Fig 1C). Conversely, the relative levels of *irf6* transcripts in embryos from wild type and paternal *irf6* ^*Δ8bp/Δ8bp*^ parents were comparable (Fig 1C). Western blot analysis using a polyclonal antibody specific for zebrafish Irf6 was performed and confirmed that Irf6 protein was undetectable in embryos from maternal *irf6* ^*Δ8bp/Δ8bp*^ crosses but comparable between embryos from wild type and paternal *irf6* ^*Δ8bp/Δ8bp*^ crosses (Fig 1C). Furthermore, all embryos from *irf6* ^*+/Δ8bp*^ heterozygous females crossed with *irf6* ^*Δ8bp/Δ8bp*^ homozygous males developed normally, when one would have expected them to rupture if the truncated Irf6 protein from the Δ8bp *irf6* allele possessed dominant-negative activity because the maternal deposition of both wild type and Δ8bp *irf6* transcripts in *irf6* ^*Δ8bp/Δ8bp*^ embryos would have led to the manifestation of the periderm rupture phenotype (Fig 1B). Taken together, the results suggest that the Δ8bp deletion caused *irf6* transcript destabilization and degradation in the oocyte cytoplasm, and significantly decreased Irf6 protein production, thus indicating that the Δ8bp *irf6* allele generated by CRISPR is a null allele (from here forward “Δ8bp” is referred to as “–“).

### Both human and zebrafish *irf6* mRNA can rescue embryonic periderm rupture and restore normal craniofacial development

**Fig 2:**
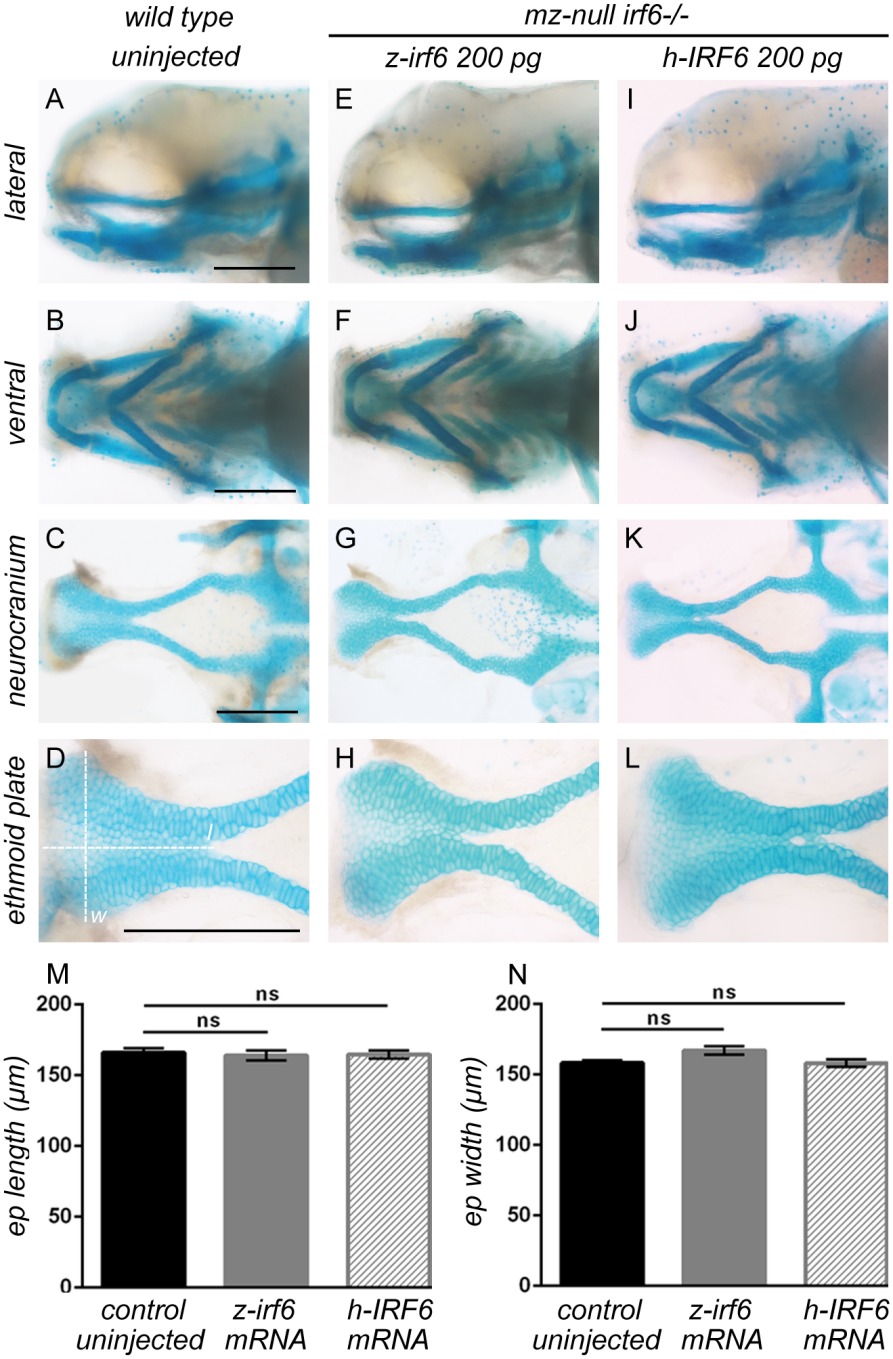
Periderm rupture and craniofacial development can be rescued by injection of either zebrafish or human wild type *IRF6* mRNA. **(A-L)** Zebrafish embryos at 96 hpf stained with alcian blue for cartilaginous craniofacial elements. Maternal/zygotic-null *irf6* ^*-/-*^ embryos were rescued by microinjection of either zebrafish *irf6* mRNA (E-H) or human *IRF6* mRNA (I-L) at the one-cell stage, preventing the periderm rupture phenotype and restoring normal craniofacial development compared to wild type embryos (A-D). Scale bar = 150 μm. **(M-N)** Dimensional measurements of dissected ethmoid plates at 96 hpf, with length (*l*) and width (*w*) denoted by dashed lines on panel (D). The length (M) and width (N) of ethmoid plates from zebrafish and human *IRF6* mRNA rescued maternal/zygotic-null *irf6* ^*-/-*^ embryos are statistically indistinguishable in dimensions compared those of wild type embryos. Error bar = 2xSEM, n = 12.

Although the CRISPR gRNA used to generate the *irf6 Δ8bp* allele was predicted *in silico* to have no off-target sites in coding regions of the zebrafish genome (crispr.mit.edu), we sought to determine the specificity of the embryonic periderm rupture phenotype from CRISPR *irf6* gene disruption by injecting wild type zebrafish *irf6* mRNA into maternal-null *irf6* ^*-/-*^ embryos at the one-cell stage, and assessing whether the periderm rupture phenotype can be rescued. Injection of zebrafish *irf6* mRNA reliably rescued the rupture phenotype in maternal-null *irf6* ^*-/-*^ embryos, and the rescued embryos were able to initiate epiboly and undergo normal embryonic development indistinguishable from wild type embryos (Fig 2A–2H).

Because of the significant sequence conservation of *IRF6* across species ranging from human to zebrafish [18], we hypothesized that it may be possible for human *IRF6* to retain its function in zebrafish. Wild type human *IRF6* cDNA (NM_006147.3) was isolated, *in vitro* transcribed into mRNA, and injected into maternal-null *irf6* ^*-/-*^ zebrafish embryos to determine whether the periderm rupture phenotype could be rescued. The ability of human *IRF6* mRNA to fully rescue the maternal-null *irf6* ^*-/-*^ periderm rupture phenotype was indistinguishable from that of zebrafish *irf6* mRNA. In addition, maternal-null *irf6* ^*-/-*^ embryos rescued by either zebrafish or human *IRF6* mRNA appeared indistinguishable from wild type embryos throughout embryonic development with normal craniofacial morphologies (Fig 2M and 2N).

### Ablation of *irf6* function during early embryogenesis significantly disrupts expression of genes critical for epithelial and craniofacial morphogenesis that can be rescued by zebrafish or human *IRF6*

**Fig 3:**
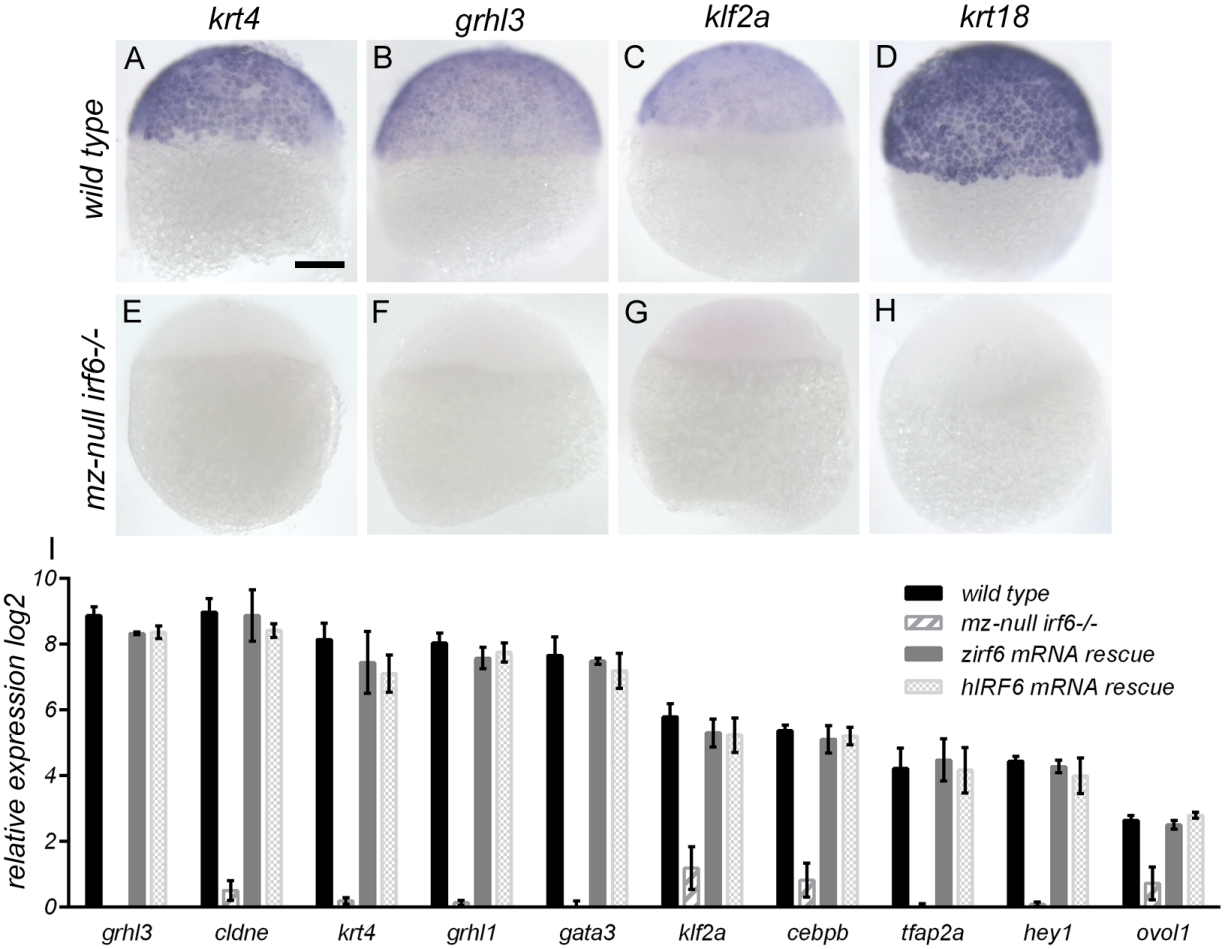
Maternal-null *irf6* ^*-/-*^ embryo gene expression is rescued by injection of either zebrafish or human wild type *IRF6* mRNA. **(A-H)** Whole-mount in situ hybridization analysis of wild type embryos (A-D) compared to maternal/zygotic-null *irf6* ^*-/-*^ embryos (E-H) at the sphere stage revealed strong down-regulation of critical *irf6* downstream genes such as *krt4* and *grhl3*. Scale bar = 150 μm. **(I)** Relative gene expression of wild type embryos, maternal/zygotic-null *irf6* ^*-/-*^ embryos, and maternal/zygotic-null *irf6* ^*-/-*^ embryos rescued with either wild type zebrafish or human *IRF6* mRNA (100 pg) microinjections, for a panel of genes with crucial roles in the *irf6* gene regulatory network. Error bar = 2xSEM, n = 3.

It was previously demonstrated that inhibition of Irf6 protein function in zebrafish causes decreased expression of several known downstream target genes such as *krt4*, *klf2a*, and *grhl3*, many of which are important for maintaining proper periderm integrity and developmental signaling pathways [23,24]. Using the *irf6* ^*-/-*^ zebrafish model, we sought to determine the gene expression changes that result from the depletion of *irf6* maternal transcripts. Whole-mount *in situ* hybridization for genes in the *irf6* gene regulatory network in maternal-null *irf6* ^*-/-*^ embryos at the sphere stage revealed significant down-regulation of gene expression in several of the genes tested, demonstrating a more complete ablation of gene expression in *irf6* downstream gene regulatory targets compared to the previously reported dominant-negative Irf6 model [22] (Fig 2A–2H). To further validate the zebrafish *irf6* ^*-/-*^ model, we sought to molecularly characterize the functional rescue of maternal-null *irf6* ^*-/-*^ periderm rupture by microinjection of zebrafish or human *IRF6* mRNA by examining the rescue of gene expression within the *irf6* gene regulatory network. RT-qPCR was performed for several genes previously known to be down-regulated in the absence of functional *irf6* such as *tfap2a* [26,27], *grhl1* [22], and *grhl3* [23,24]. The results revealed significant down-regulation of these genes in maternal-null *irf6* ^-/-^ embryos at 4 hpf compared to wild type, but statistically indistinguishable levels compared to wild type in maternal-null *irf6* ^-/-^ embryos microinjected with either zebrafish or human *IRF6* mRNA (Fig 3I). Furthermore, the qPCR results revealed significant down-regulation of genes important for epithelial and craniofacial development in maternal-null *irf6* ^*-/-*^ embryos compared to wild type (Fig 3I), suggesting that *irf6* maternal transcripts might play key roles in activating the molecular pathways required for zebrafish periderm maturation and epiboly, and potentially later for signaling pathways related to craniofacial development.

### PolyPhen-2 and SIFT predictions of *IRF6* variant protein function do not accurately reflect their ability to rescue zebrafish periderm rupture

**Fig 4:**
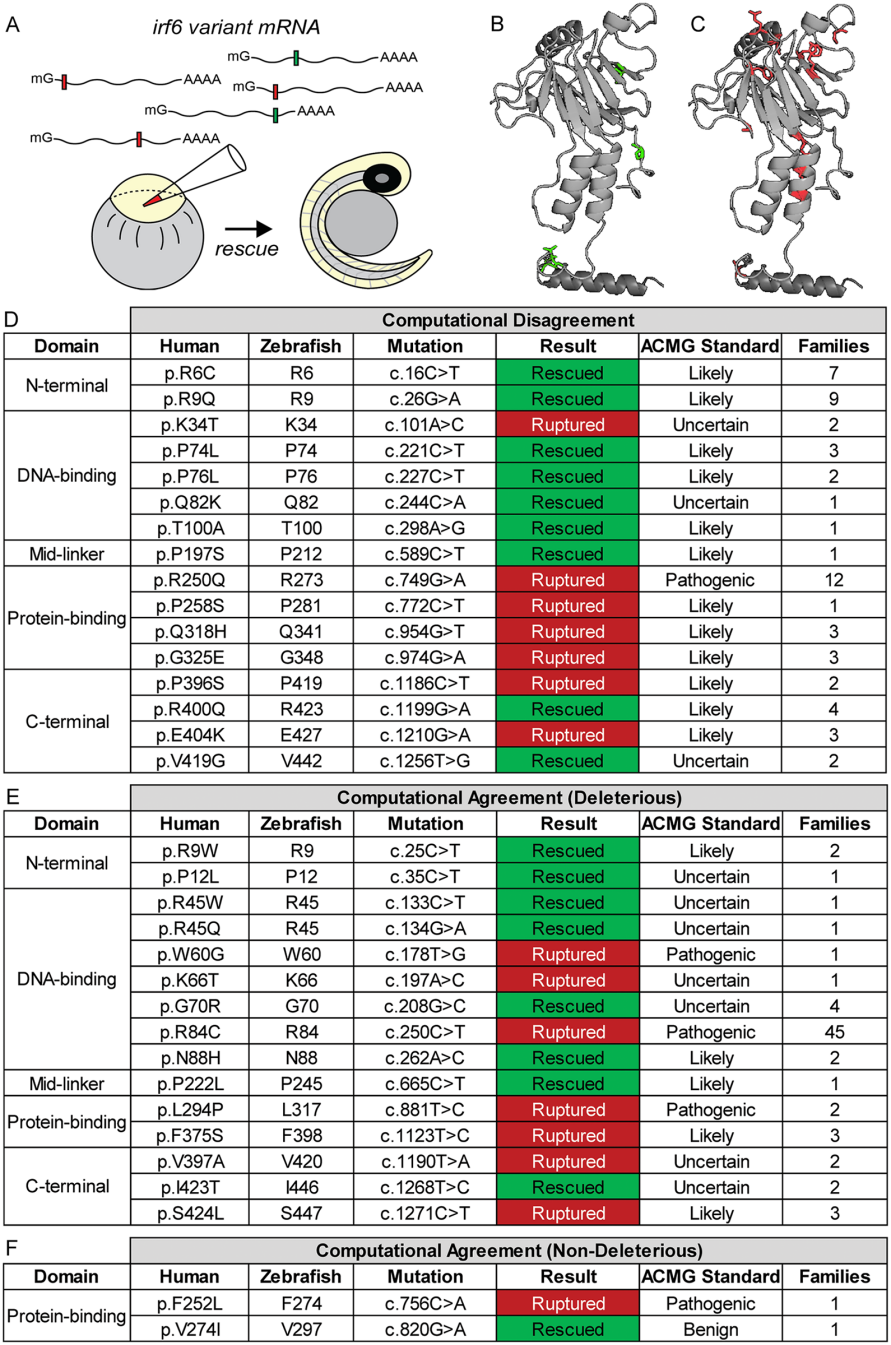
Functional characterization of human *IRF6* missense gene variant protein functions with the zebrafish *irf6* model. **(A)** Experimental approach for characterizing protein functions of human *IRF6* missense gene variants. Variant mRNAs were synthesized and microinjected into maternal-null *irf6* ^*-/-*^ embryos at the one-cell stage and assessed for phenotypic rescue at 24 hpf. **(B-C)** Protein modeling of the protein-binding domain and C-terminus of IRF6 using ExPASy with crystalline structures of IRF1. (B) is mapped with missense variant amino acid residues (green) whose mRNA rescued the periderm rupture phenotype, while (C) is mapped with missense variant amino acid residues (red) whose mRNA failed to rescue. **(D-F)** Results for functional rescue of periderm rupture with maternal-null *irf6* ^*-/-*^ embryos for representative human *IRF6* missense gene variants. Results were classified as rescued if any maternal-null *irf6* ^*-/-*^ embryos injected with variant mRNA remained alive and phenotypically wild type at 24 hpf (50 embryos/round, n = 3). Missense gene variants were categorized by location within the IRF6 protein, and by computational results from PolyPhen-2 and SIFT on whether the *in silico* predictions agreed on the deleterious effects of the missense gene variants on protein function. Further shown are ACMG guideline pathogenicity predictions (pathogenic, likely pathogenic, uncertain, and benign), and the number of families identified for each variant (all gene variant annotations were based on NM_006147.3).

The conservation of human *IRF6* protein function in zebrafish provided an opportunity to assess the protein functions of human *IRF6* missense gene variants. Over 300 *IRF6* gene variants have been identified from human CL/P patients and approximately 50% are missense variants [15]. Two of the more commonly used computational prediction programs, PolyPhen-2 [28] and SIFT [29], were used to predict the effects of amino acid substitutions on the protein functions of *IRF6* missense variants and segregate them into three categories: 1) both programs agree the variant disrupts protein function, resulting in a loss-of-function protein, 2) the programs disagree on the effects of the variant on protein function, and 3) both programs agree the variant does not disrupt protein function. Human *IRF6* missense gene variants were then mapped to their corresponding nucleotides in the zebrafish *irf6* cDNA by sequence conservation, *in vitro* transcribed into mRNA, and microinjected into maternal-null *irf6* ^*-/-*^ zebrafish embryos to assess their ability to rescue the periderm rupture phenotype (Fig 4A). The results demonstrated that the computational programs did not offer a significant advantage in predicting the biological functions of variant Irf6 proteins (Fig 4D–4F). Variants that received conflicting predictions from PolyPhen-2 and SIFT also provided mixed results in their abilities to rescue (Fig 4D). Moreover, variants that were predicted by both computational programs to result in loss-of-function proteins were often able to experimentally rescue the rupture phenotype (Fig 4E). Lastly, missense gene variants that were predicted by both programs to be non-deleterious to protein function were also mixed in their abilities to rescue, further demonstrating the limitations of these computational programs at predicting protein function (Fig 4F). For example, the missense gene variant p.F252L that was predicted by both programs to be non-deleterious to protein function was unable to rescue the maternal-null *irf6* ^*-/-*^ rupture phenotype (Fig 4F). When the experimentally tested *IRF6* gene variants were grouped according to their abilities to rescue periderm rupture and mapped to the predicted human IRF6 protein structure (protein-binding domain and C-terminus) generated by ExPASy, the distribution of the amino acid residues suggested variants that could not rescue mostly resided in protein secondary structures and thus likely to disrupt protein conformation and function (Fig 4C). Conversely, amino acid residues for human *IRF6* variants that retained protein function and rescued periderm rupture mostly mapped to regions without secondary structures (Fig 4B) and thus less likely to disrupt protein conformations critical for IRF6 function. In addition, the variants tested were re-examined for their genetic backgrounds and the numbers of individuals previously identified with the variant and this information was used to classify the variants according to the five-category system for variant pathogenicity established by the American College of Medical Genetics guidelines [30] (Fig 4D–4F). While no variants in the highest pathogenicity certainty category of “Pathogenic” were able to rescue the rupture phenotype, other variants with varying degrees of uncertainty in pathogenicity were mixed in their abilities to rescue (Fig 4D–4F). The only variant identified to be classified as “Benign”, p.V274I, was able to rescue the rupture phenotype (Fig 4F).

### Dosage titrations can differentiate functional categories of human *IRF6* missense gene variants

**Fig 5:**
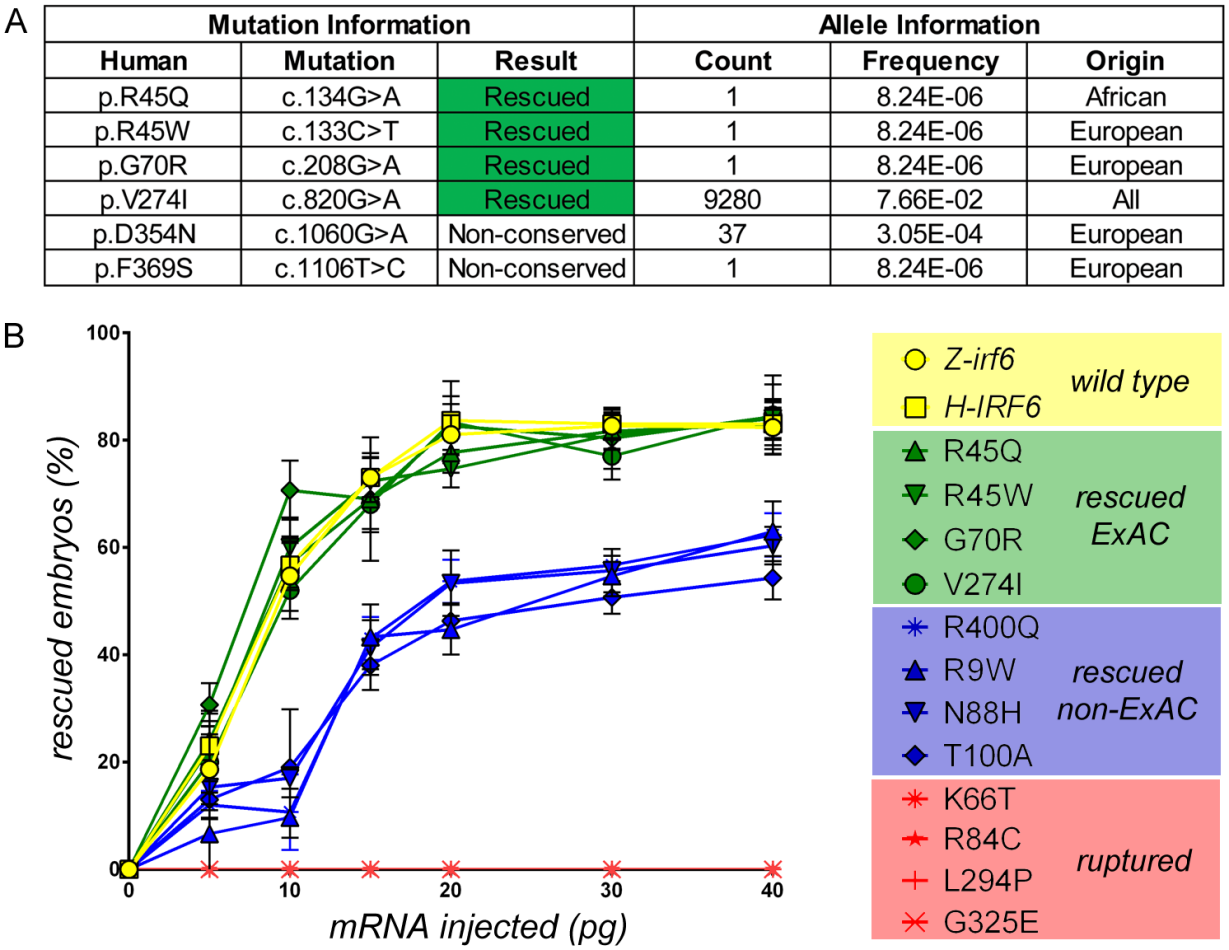
Zebrafish periderm rupture assay can detect *IRF6* missense gene variants with reduced protein function by mRNA dosage titration. **(A)** Identification and characterization of *IRF6* missense gene variants in the ExAC and gnomAD databases. p.V274I alleles were identified in all populations. **(B)** mRNA dosage titration experiement results for a subset of missense gene variants correlating amount of variant mRNA microinjected to the percent of maternal-null *irf6* ^*-/-*^ embryos rescued from rupture and undergoing normal embryonic development at 24 hpf. The missense variants were classified into three categories based on levels of protein function. No variant was identified in ExAC/gnomAD that could not rescue the periderm rupture phenotype. Error bar = 2xSEM, 50 embryos/round, n = 3.

The human *IRF6* missense gene variants functionally tested in this study could result in reduced function rather than complete loss-of-function proteins, thereby leaving open the possibility that while they can rescue the zebrafish maternal-null *irf6* ^*-/-*^ rupture phenotype in this assay, their reduced function *in vivo* is sufficient to cause disease in humans. To address this possibility, the mRNA of wild type zebrafish *irf6*, human *IRF6*, and several missense gene variants were individually microinjected into maternal-null *irf6* ^*-/-*^ embryos at the one-cell stage through a range of concentrations to establish a titration response curve for periderm rupture rescue (Fig 5B). To assist in the functional analysis of these *IRF6* missense gene variants with potentially more nuanced functional changes, we utilized the significantly increased statistical power of rare human gene variant detection provided by the Exome Aggregation Consortium database of over 60,000 individuals [10,15]. In addition, we also examined the gnomAD database, a more recent effort from the same group which now extended to over 125,000 exomes and 15,000 whole genomes. From these databases, the *IRF6* missense gene variants p.R45Q, p.R45W, p.G70R, p.V274I, p.D354N, and p.F369S were identified. Through various lines of evidence, the variant p.V274I was already considered benign by ACMG standards (Fig 5A). Indeed, 9,280 alleles of this gene variant (allele frequency 0.077) was found in ExAC distributed across all populations including Europeans, Africans, and Asians. p.D354N and p.F369S were found to be non-conserved in zebrafish *irf6* and illustrates the possibility that the identities of these residues are not essential for IRF6 protein function. In addition, although p.D354N was previously identified in four VWS pedigrees, 37 alleles were also found in the ExAC/gnomAD database. Three other missense gene variants, p.R45Q, p.R45W, and p.G70R were identified as single alleles in the ExAC/gnomAD database and were able to rescue the periderm rupture phenotype of maternal-null *irf6* ^*-/-*^ embryos. The small number of these alleles do not provide as strong of support for non-pathogenicity as p.V274I. But, their presence in ExAC/gnomAD does raise the possibility that these gene variants could retain protein function and potentially be non-pathogenic, and therefore should be functionally tested. The aforementioned variants, in addition to p.V274I and several others that were mixed in their ability to rescue were used in a mRNA microinjection dosage titration assay. Interestingly, the tested missense gene variants naturally segregated into three functional categories upon dosage titration. The variants identified in ExAC/gnomAD rescued to the same degree as wild type zebrafish and human *IRF6* mRNA (Fig 5B Green). Variants that were able to rescue in the periderm rupture assay but not found in ExAC/gnomAD were found to be reduced in protein function compared to wild type, rescuing a smaller percentage of embryos at each of the mRNA dosages tested (Fig 5B Blue). These results are in contrast to the missense gene variants that could not rescue the rupture phenotype at any dosage tested, which were not found in ExAC/gnomAD or other public exome/genome databases, suggesting that they are complete loss-of-function variant proteins and likely pathogenic in human orofacial cleft patients.

### Human *IRF6* gene variants are capable of restoring zebrafish embryonic development

**Fig 6:**
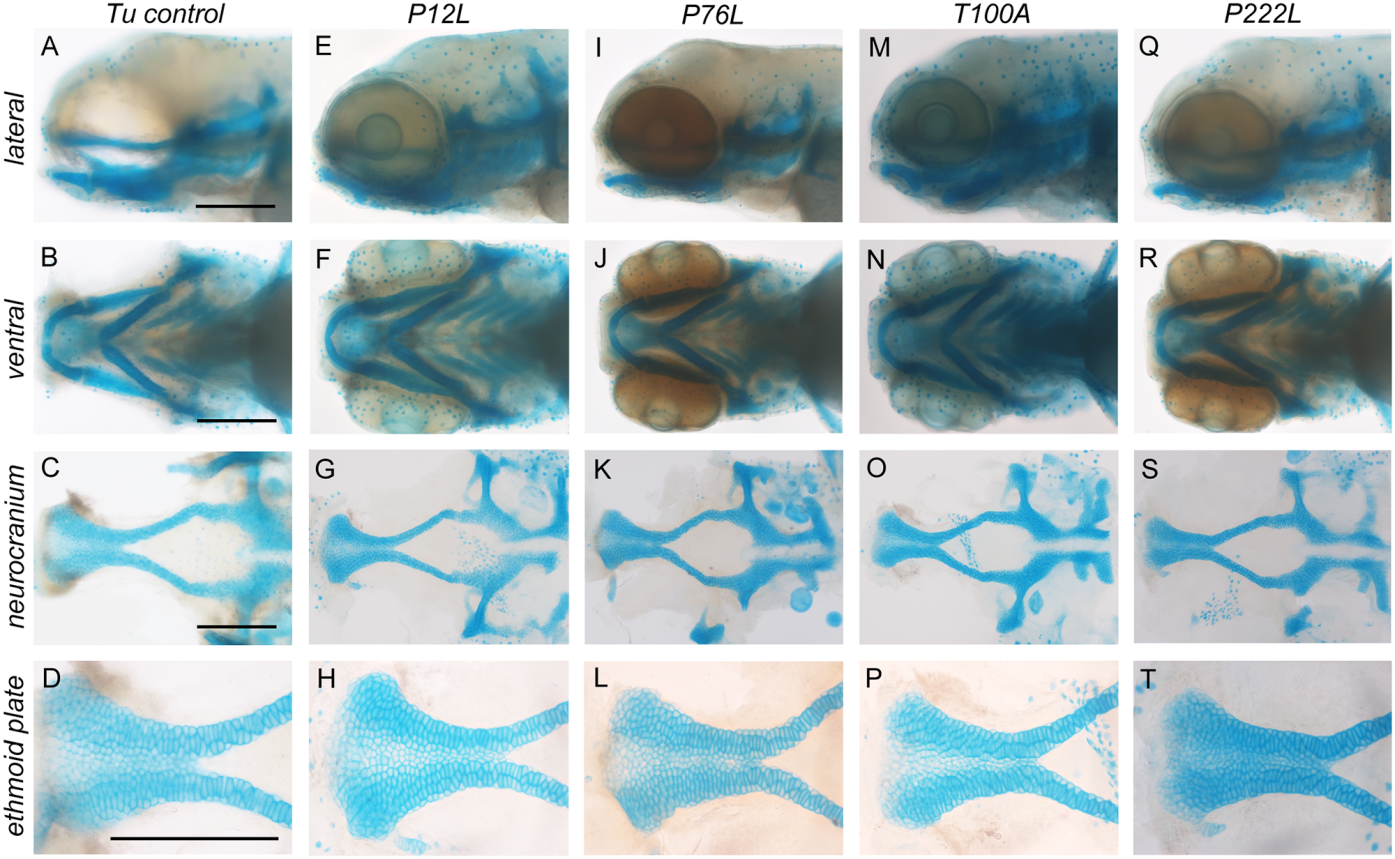
*IRF6* missense gene variants can rescue periderm rupture and restore normal craniofacial development. **(A-T)** Craniofacial morphologies of maternal-null *irf6* ^*-/-*^ embryos rescued by human *IRF6* missense gene variant mRNA microinjections (100 pg/embryo) at 96 hpf stained with alcian blue. (A-D) Uninjected wild type control. (E-H) p.P12L. (I-L) p.P76L. (M-P) p.T100A. (Q-T) p.P222L. Scale bars = 150 μm, n = 3.

Although the *IRF6* missense gene variants that were not found in ExAC/gnomAD had reduced protein activities, they otherwise retained their biological functions and were not only able to rescue the zebrafish periderm rupture phenotype at high mRNA injection dosages, but also permitted normal embryonic development (Fig 6). The variant mRNA-rescued maternal-null *irf6* ^*-/-*^ embryos that otherwise would have ruptured were grown under standard conditions and resulted in not only phenotypically wild type craniofacial development (Fig 6), but also in viable and fertile adults.

## Discussion

### Challenges in statistical and computational analyses of rare human *IRF6* missense gene variants

While it is tremendously useful to document human genetic variations in genes associated with disease from a wide range of populations, as in the case of *IRF6* for orofacial clefts, a key challenge in human genetics remains how to functionally ascertain whether coding sequence variations result in harmful alterations in protein function, and whether these functional changes are pathogenic for disease [5,6]. Statistical methods can provide support for the pathogenicity of gene variants by examining the segregation of pathogenic variants with disease status within affected families, or by examining the frequencies of the variants in the population or disease cases versus control [8]. However, such statistical methods do not test biological protein functions and are prone to biases, especially for rare gene variants. Distinct but unobserved pathogenic variants may be located on the same haplotype as the candidate rare variant and thus segregation analysis alone cannot unambiguously assign pathogenicity, especially in smaller pedigrees [1]. In addition, the incidence of CL/P is higher in regions of the world where whole exome and genome sequencing has not yet captured a large cross section of the normal population for use as controls. Due to this geographical clustering, many cases of newly discovered *IRF6* gene variants cannot be statistically compared to public exome/genome databases because the population mismatch could over emphasize the relative rarity of certain gene variants and thereby their potential pathogenicity.

Computational programs such as PolyPhen-2 and SIFT that predict the effects of missense mutations on variant protein functions use complex algorithms that take into consideration a multitude of parameters to predict the thermodynamic stability and functions of variant proteins after amino acid substitutions. However, there are still many unaccounted factors that go into translating amino acid changes to changes in protein function and thus such computational programs often provide results that conflict with biological evidence. Since many computational prediction programs depend on machine-learning algorithms [31], the direct biological assessment of variant protein functions could be reiterated through the same algorithms to improve their predictive powers for both IRF6 and other proteins with similar sequences and motifs. According to the recent ACMG guidelines, the assessment of gene variant pathogenicity should be multi-pronged with various independent lines of supporting evidence from different approaches, including statistical, computational and experimental [30]. While pathogenicity assignments typically cannot be made from any line of evidence alone, using experimental models to directly interrogate variant protein functions provide valuable insights into the biological effects of missense variant amino acid substitutions on protein functions and can greatly aid in the interpretation of variant protein function and pathogenicity.

### Zebrafish periderm as a model of mammalian oral epithelium and the requirement of maternal *irf6* during epiboly and craniofacial development

The zebrafish *irf6* ^*-/-*^ model revealed the importance of *irf6* maternal transcripts for embryonic periderm development, corroborating previous reports that characterized *irf6* function in the zebrafish and *Xenopus laevis* models [22,23]. However, in contrast to previous studies of *irf6* performed using mRNA injections of dominant-negative Irf6 which could result in delayed and incomplete knockdown of Irf6 function, the genetic disruption of *irf6* reported in this study more completely ablated Irf6 function in the early embryo with near undetectable expression of downstream transcriptional targets such as *grhl3* and *krt4* [24,25,32,33]. This *irf6* null model exhibited developmental arrest at the sphere stage and suggests that *irf6* is a potential epiboly initiation factor necessary for regulating cell signaling pathways that orchestrate this complex morphological event during zebrafish embryogenesis. Interestingly, paternal-null *irf6* ^*-/-*^ embryos developed normally into adulthood, suggesting that zygotic transcription of *irf6* may not be necessary in zebrafish for normal embryonic development, possibly due to the persistence of maternal Irf6 protein throughout early embryogenesis. *Irf6* ^*-/-*^ mice exhibited cleft palates with oral epithelial adhesions and a number of other epithelial abnormalities, suggesting that *IRF6* plays an important role in regulating epithelial proliferation and differentiation [19,20]. The zebrafish embryonic periderm has emerged as a model of the mammalian oral epithelium because many of the gene regulatory networks and cellular behaviors during zebrafish epiboly, such as convergence-extension and epithelial-to-mesenchymal transition, are conserved in the mammalian oral epithelium during palate development [24,25,34]. The observation that maternal-null *irf6* ^*-/-*^ embryos failed to initiate epiboly cellular movements suggests that many of the downstream pathways and cellular behaviors are dependent upon the proper establishment of the periderm, and emphasizes the potential importance of the oral epithelium in initiating and orchestrating both epithelial and mesenchymal tissue behaviors during palate development in the mammalian system. Because of the early embryonic lethal periderm rupture phenotype of maternal-null *irf6* ^*-/-*^ embryos, we are in the process of elucidating the functional requirements of Irf6 during craniofacial development in zebrafish, separated from its functions in the establishment of the periderm during epiboly.

### Functional rescue of zebrafish periderm rupture with *IRF6* missense gene variants

The experimental finding that human *IRF6* mRNA could rescue not only periderm rupture in zebrafish maternal-null *irf6* ^*-/-*^ embryos but also normal embryonic development and *IRF6* gene regulatory network gene expression suggests that there is significant cross-species conservation in IRF6 protein structure and function. Taken together, our finding demonstrated that the zebrafish maternal-null *irf6* ^*-/-*^ model could serve as a sensitive and specific platform for the rapid assessment of human *IRF6* missense variant protein functions in a relevant *in vivo* context. This biological assay can complement the statistical analyses to form a more comprehensive picture in the process of assigning pathogenicity to *IRF6* missense gene variants. This complementary approach is especially important for rare *IRF6* gene variants identified in a small number of individuals often in a single pedigree. In the case of p.R45W, this gene variant was detected in a single VWS affected proband but also in his unaffected sibling and mother, leaving in question whether this missense variant is truly pathogenic or simply a rare benign variant that was annotated as pathogenic despite the imperfect variant co-segregation with disease which was previously attributed to incomplete penetrance and variable expressivity [35]. Although this interpretation is possible due to the variable expressivity of phenotypes exhibited by VWS patients in the same pedigree, other interpretations are possible such as a case where an unobserved pathogenic variant in a separate gene adjacent to *IRF6* is on the same haplotype co-segregating with the p.R45W variant. This interpretation is further supported by the biological analysis of p.R45W variant protein function in the zebrafish model, which revealed that the p.R45W variant protein was able to rescue maternal-null *irf6* ^*-/-*^ periderm rupture and function quantitatively to the same degree as wild type *IRF6* (Fig 5B). While this experimental validation of p.R45W variant protein function does not conclusively exclude its potential pathogenicity in human patients, it does suggest that further biological evidence is needed before p.R45W can be annotated as pathogenic in public databases and used in the clinical diagnosis of VWS patients.

The functional genomics validation of *IRF6* missense gene variant protein function presented in this study is complemented by rapid increases in statistical power for rare gene variant identification and pathogenicity assignment through expansions in large public exome/genome databases. More and more individuals are sequenced daily with advances in sequencing technologies and concomitantly decreasing sequencing costs. Several *IRF6* missense gene variants previously unobserved in the general population and therefore thought to be pathogenic were identified in the ExAC/gnomAD databases [10], potentially weakening the statistical power of their pathogenicity assignments by ACMG standards. In addition, their discovery in the ExAC/gnomAD databases streamlined the variant protein functional validation process by identifying variants with the highest probability of ambiguous pathogenicity assignments for testing in our model. The zebrafish maternal-null *irf6* ^*-/-*^ model allowed for the detection of subtle changes in *IRF6* protein function such as reduced function variants through mRNA microinjection dosage titrations. The stability of variant mRNA and proteins could also be readily assessed through molecular biology techniques after mRNA microinjections. The missense variants p.R45Q, p.R45W, p.G70R, and p.V274I were able to not only rescue the periderm rupture phenotype of maternal-null *irf6* ^*-/-*^ zebrafish embryos, but were also functionally indistinguishable from wild type zebrafish and human *IRF6* in dosage titration, suggesting that the proteins produced from these missense variants retained full function. However, the pathogenicity of these variants cannot be conclusively determined with this *IRF6* functional model due to the possibility that only a subset of *IRF6* functions are conserved between human and zebrafish. Interestingly, the variants tested that were able to rescue in the embryo rupture assay and yet not found in the ExAC/gnomAD databases were reduced in protein function when compared to wild type zebrafish or human *IRF6*. This result suggests that these missense gene variants are not complete loss-of-function variants but rather reduced in function, and thus still potentially pathogenic in humans because their reduced functions might be insufficient to prevent phenotype and disease onset. Lastly, the variants that were not able to rescue the maternal-null *irf6* ^*-/-*^ periderm rupture at any of the dosages tested likely represent complete loss-of-function missense variants. These variants were not found in the ExAC/gnomAD databases, and their complete loss-of-function provide an additional line of biological evidence in support for their pathogenic status in human VWS patients. Because of the relatively small number of missense gene variants functionally tested through dosage titration in this study, no variant was discovered to be functionally wild type and yet not identified in the ExAC/gnomAD databases. Although the collection of sequenced exomes and genomes is continuously increasing, a larger control population still does not guarantee the discovery of rare gene variants previously thought to be pathogenic in human disease. In these situations, experimental validation of rare gene variant protein functions could bridge a gap in knowledge and provide additional biological insights to assist in variant pathogenicity assignments and improve their accuracies. While it is possible that only a subset of *IRF6* protein functions are conserved in the zebrafish model and tested through this functional rescue assay, this model serves to provide novel insights into the effects of human *IRF6* missense gene variants on IRF6 protein function, and complement current statistical and bioinformatics results.

Overall, the zebrafish maternal-null *irf6* ^*-/-*^ model not only offers a method to rapidly assess the protein functions of current and yet undiscovered human *IRF6* missense gene variants, but also illustrates a generalizable functional genomics paradigm where novel human gene variants can be biologically tested for protein function using corresponding zebrafish mutants to provide another line of biological evidence to assist with pathogenicity assignments. With advances in CRISPR-Cas9 targeted mutagenesis in zebrafish, it will be increasingly efficient to develop zebrafish gene disruption and rescue assays to test the functions of human gene variants for genes in other contexts of disease.

## Materials and methods

### Ethics statement

All work with zebrafish (adult, larval, and embryonic) was performed in strict accordance with protocols approved by Massachusetts General Hospital IACUC (Protocol 2010N000106).

### Fish rearing and husbandry

Zebrafish *Danio rerio* were maintained in accordance with approved institutional protocols at Massachusetts General Hospital, as described [36]. Embryos were kept at 28.5°C in E3 media containing 0.0001% methylene blue and staged according to [37] by hours or days post fertilization (hpf or dpf). All wild type and mutant zebrafish lines used in these experiments were generated from the Tübingen (Tü) strain.

### CRISPR gene editing and genotyping

Potential CRISPR gRNA target sites were identified using the CRISPR design program at (zifit.partners.org/ZiFiT/ and crispr.mit.edu). The *irf6* exon 6 gRNA was generated by in vitro transcription from a T7 promoter as described [38]. Zebrafish optimized cas9 template DNA pT3TS-nls-zCas9-nls [39] was linearized using XbaI and purified using the QIAquick PCR purification kit (Qiagen). Capped *cas9* mRNA was synthesized from a T3 promoter using the mMESSAGE mMACHINE T3 transcription kit (Ambion) and purified using the RNeasy mini kit (Qiagen). One-cell staged zebrafish embryos were injected directly in the cytoplasm with 2 nl of a solution containing 25 ng/μl of gRNA and 100 ng/μl of *cas9* mRNA. Genomic DNA for genotyping was isolated from either whole 24 hpf embryos or tail fin clips using the HotSHOT method [40]. Genotyping primers flanking CRISPR target site were designed with a forward primer modified with 5’-FAM and submitted for microsatellite analysis to determine indel mutation size and frequencies. Sanger sequencing of the CRISPR target site was performed by cloning the genotyping PCR amplicon into pGEM-T easy (Promega) to validate the exact sequence changes from CRISPR mutagenesis.

### RNA isolation and quantitative RT-PCR

Stage-matched zebrafish embryos were flash-frozen in liquid nitrogen and homogenized with a micropestle in TRIzol (Invitrogen). Total RNA was isolated using phenol-chloroform exaction and digested using DNase I (Ambion) to remove genomic DNA contamination. Total RNA was quantified using a NanoDrop spectrophotometer and 5 μg was used for reverse transcription using the SuperScript III cDNA synthesis kit (Invitrogen). Quantitative RT-PCR was performed using PowerUP SYBR Green qPCR master mix (Invitrogen) on the StepOne Plus RT-PCR platform (Applied Biosystems). Elongation factor 2α or β-actin were used as internal controls for expression normalization. Amplification specificity was checked using melt-curve analysis. Control amplifications were performed on samples either without reverse transcription or template.

### Protein isolation and western blot

Zebrafish embryos were enzymatically dechorionated with pronase (Sigma) and deyolked according to [41] supplemented with HALT protease inhibitor cocktail (Thermo Scientific). Cell pellets were flash frozen in liquid nitrogen and homogenized using a micropestle in RIPA buffer. Protein lysate concentrations were quantified using the DC Protein Assay kit (Bio-Rad) and subjected to electrophoresis (20 μg/lane) in Novex Bis-Tris 10% protein gels (Invitrogen). Gels were subsequently blotted onto a 0.22 μm PVDF membrane (Novex), blocked for two hours at room temperature with StartingBlock in TBST (Thermo Scientific) and incubated with a rabbit polyclonal antibody for zebrafish Irf6 (GeneTex) (1:1500 dilution) and a rabbit monoclonal antibody for zebrafish ß-actin (Cell Signaling Technology) (1:1500 dilution) in blocking buffer at 4°C overnight. The membrane was washed 3x 10 min at room temperature and incubated with a HRP-conjugated anti-rabbit antibody (Abcam) (1:2000 dilution) in blocking buffer at room temperature for one hour. Bands were visualized using Novex ECL chemiluminescence reagent (Invitrogen).

### *IRF6* cDNA cloning and variant generation

Full-length zebrafish *irf6* (NM_200598.2) and human *IRF6* (NM_006147.3) cDNA was synthesized using GeneArt (Invitrogen) and sub-cloned into the pCS2+8 vector (Addgene #34931) at the EcoRV site in the MCS. *IRF6* gene variants were identified in previously published literature and mapped to their corresponding nucleotides in the zebrafish *irf6* cDNA. PCR-based site-directed mutagenesis was performed to generate pCS2+8 vectors containing *irf6* missense gene variants using the Q5 site-directed mutagenesis kit (New England Biolabs) with amplification primers designed by the NEBaseChanger online tool (http://nebasechanger.neb.com). Plasmids containing *irf6* missense gene variants were isolated using the QIAprep spin miniprep kit (Qiagen) and sequence verified with either complete plasmid sequencing or Sanger sequencing through the entire cDNA region.

### Variant mRNA synthesis and embryo microinjections

Plasmids (pCS2+8 backbone) containing individual *IRF6* missense gene variant cDNAs were linearized by NotI-HF and purified using QIAquick PCR purification columns (Qiagen). Variant mRNA was synthesized by in vitro transcription using the SP6 mMESSAGE mMACHINE transcription kit (Ambion) and linearized plasmids as template. cDNA template was digested using DNase I and variant mRNA was purified using the RNeasy mini kit (Qiagen) and quantified with either a NanoDrop spectrophotometer or Qubit fluorometer (Invitrogen). Variant mRNAs were diluted to 800 ng/μl and stored as aliquots in -80°C. For embryo microinjections, 2 nl of the injection mix was delivered directly into the cytoplasm of one-cell staged embryos with variant mRNA diluted to a final concentration of 50 ng/μl.

### Whole-mount in situ hybridization

Zebrafish embryos were fixed in 4% paraformaldehyde (PFA) at 4°C overnight and subsequently transferred into 100% methanol prior to whole-mount in situ hybridization (WISH). WISH and DIG-labeled riboprobe synthesis were performed essentially as described in [42]. All riboprobes were amplified from mix-staged embryonic zebrafish cDNA, cloned into pGEM-T easy (Promega), and direction/sequence verified with Sanger sequencing. Riboprobes were synthesized from either the T7 or SP6 promoter using a digoxigenin (DIG) labeling kit (Roche). Detection was performed with an alkaline phosphatase conjugated anti-DIG antibody (Roche) and BCIP/NBT colorimetric substrates (Sigma).

### Alcian blue staining and imaging

Zebrafish embryos were fixed in 4% PFA at 4°C overnight and bleached (0.8% W/V KOH, 0.1% Tween20, 0.9% H_2_O_2_) until pigmentation of cells were no longer present. Acid-free alcian blue staining was performed essentially as described [42] overnight on a rotating platform at room temperature. Whole or dissected stained embryos were mounted in 3% methylcellulose on a depression slide and imaged using a Nikon Eclipse 80i compound microscope with a Nikon DS Ri1 camera. Z-stacks were taken to increase the depth-of-field using the NIS Element BR 3.2 software and processed by ImageJ to provide a composite maximum intensity projection image.

### Computational modeling and statistical analyses

Human *IRF6* missense gene variants were identified from previously published literature [15] and entered into PolyPhen-2 (http://genetics.bwh.harvard.edu/pph2) and SIFT (http://sift.jcvi.org) for computational predictions of missense variant protein function. The ExPASy SWISS-MODEL online tool (https://swissmodel.expasy.org) was used to align the IRF6 amino acid sequence with the known crystalline structure of IRF1 to model the IRF6 SMIR/IAD protein-binding domain and C-terminus. The resulting protein structures were visualized using PyMOL. For statistical analysis of experimental data, error bars represent ±2x standard mean error (SEM), and statistical significance was interrogated using two-tailed Student’s T-tests with <0.05 as the P-value cut-off. For the identification of *IRF6* missense variants from the ExAC database (exac.broadinstitute.org) and gnomAD database (gnomad.broadinstitute.org), *IRF6* was queried and the results were sorted for missense gene variants.

## References

[1] MacArthur DG, Manolio TA, Dimmock DP, Rehm HL, Shendure J, et al (2014) Guidelines for investigating causality of sequence variants in human disease. Nature 508: 469–476. doi: 10.1038/nature13127 24759409 10.1038/nature13127 PMC4180223

[2] Durbin RM, Abecasis GR, Altshuler DL, Auton A, Brooks LD, et al (2010) A map of human genome variation from population-scale sequencing. Nature 467. 10.1038/nature09534 PMC3042601 20981092

[3] König E, Rainer J, Domingues FS (2016) Computational assessment of feature combinations for pathogenic variant prediction. Molecular Genetics & Genomic Medicine 4: 431–446. 27468419 10.1002/mgg3.214 PMC4947862

[4] Rasmussen LJ, Heinen CD, Royer-Pokora B, Drost M, Tavtigian S, et al (2012) Pathological assessment of mismatch repair gene variants in Lynch syndrome: Past, present, and future. Human Mutation 33: 1617–1625. doi: 10.1002/humu.22168 22833534 10.1002/humu.22168

[5] Stitziel NO, Kiezun A, Sunyaev S (2011) Computational and statistical approaches to analyzing variants identified by exome sequencing. Genome Biology 12: 1–10. 10.1186/gb-2011-12-9-227 PMC3308043 21920052

[6] Manolio TA, Collins FS, Cox NJ, Goldstein DB, Hindorff LA, et al (2009) Finding the missing heritability of complex diseases. Nature 461: 747–753. doi: 10.1038/nature08494 19812666 10.1038/nature08494 PMC2831613

[7] Thusberg J, Olatubosun A, Vihinen M (2011) Performance of mutation pathogenicity prediction methods on missense variants. Human Mutation 32: 358–368. doi: 10.1002/humu.21445 21412949 10.1002/humu.21445

[8] Wei Q, Wang L, Wang Q, Kruger WD, Dunbrack RL (2010) Testing computational prediction of missense mutation phenotypes: Functional characterization of 204 mutations of human cystathionine beta synthase. Proteins: Structure, Function, and Bioinformatics 78: 2058–2074. 10.1002/prot.22722 PMC3040297 20455263

[9] Bell CJ, Dinwiddie DL, Miller NA, Hateley SL, Ganusova EE, et al (2011) Carrier Testing for Severe Childhood Recessive Diseases by Next-Generation Sequencing. Science Translational Medicine 3: 65ra64. 10.1126/scitranslmed.3001756 PMC3740116 21228398

[10] Lek M, Karczewski KJ, Minikel EV, Samocha KE, Banks E, et al (2016) Analysis of protein-coding genetic variation in 60,706 humans. Nature 536: 285–291. doi: 10.1038/nature19057 27535533 10.1038/nature19057 PMC5018207

[11] Quintáns B, Ordóñez-Ugalde A, Cacheiro P, Carracedo A, Sobrido MJ (2014) Medical genomics: The intricate path from genetic variant identification to clinical interpretation. Applied & Translational Genomics 3: 60–67. 27284505 10.1016/j.atg.2014.06.001 PMC4887840

[12] Kondo S, Schutte BC, Richardson RJ, Bjork BC, Knight AS, et al (2002) Mutations in IRF6 cause Van der Woude and popliteal pterygium syndromes. Nat Genet 32: 285–289. doi: 10.1038/ng985 12219090 10.1038/ng985 PMC3169431

[13] de Lima RLLF, Hoper SA, Ghassibe M, Cooper ME, Rorick NK, et al (2009) Prevalence and nonrandom distribution of exonic mutations in interferon regulatory factor 6 in 307 families with Van der Woude syndrome and 37 families with popliteal pterygium syndrome. Genet Med 11: 241–247. doi: 10.1097/GIM.0b013e318197a49a 19282774 10.1097/GIM.0b013e318197a49a PMC2789395

[14] Little HJ, Rorick NK, Su LI, Baldock C, Malhotra S, et al (2009) Missense mutations that cause Van der Woude syndrome and popliteal pterygium syndrome affect the DNA-binding and transcriptional activation functions of IRF6. Hum Mol Genet 18: 535–545. doi: 10.1093/hmg/ddn381 19036739 10.1093/hmg/ddn381 PMC2638798

[15] Leslie EJ, Standley J, Compton J, Bale S, Schutte BC, et al (2013) Comparative analysis of IRF6 variants in families with Van der Woude syndrome and popliteal pterygium syndrome using public whole-exome databases. Genet Med 15: 338–344. doi: 10.1038/gim.2012.141 23154523 10.1038/gim.2012.141 PMC3723330

[16] Rahimov F, Jugessur A, Murray JC (2012) Genetics of nonsyndromic orofacial clefts. Cleft Palate Craniofac J 49: 73–91. doi: 10.1597/10-178 21545302 10.1597/10-178 PMC3437188

[17] Zucchero TM, Cooper ME, Maher BS, Daack-Hirsch S, Nepomuceno B, et al (2004) Interferon Regulatory Factor 6 (IRF6) Gene Variants and the Risk of Isolated Cleft Lip or Palate. New England Journal of Medicine 351: 769–780. doi: 10.1056/NEJMoa032909 15317890 10.1056/NEJMoa032909

[18] Ben J, Jabs EW, Chong SS (2005) Genomic, cDNA and embryonic expression analysis of zebrafish IRF6, the gene mutated in the human oral clefting disorders Van der Woude and popliteal pterygium syndromes. Gene Expr Patterns 5: 629–638. doi: 10.1016/j.modgep.2005.03.002 15939375 10.1016/j.modgep.2005.03.002

[19] Ingraham CR, Kinoshita A, Kondo S, Yang B, Sajan S, et al (2006) Abnormal skin, limb and craniofacial morphogenesis in mice deficient for interferon regulatory factor 6 (Irf6). Nat Genet 38: 1335–1340. 17041601 10.1083/ng1903 PMC2082114

[20] Richardson RJ, Dixon J, Malhotra S, Hardman MJ, Knowles L, et al (2006) Irf6 is a key determinant of the keratinocyte proliferation-differentiation switch. Nat Genet 38: 1329–1334. doi: 10.1038/ng1894 17041603 10.1038/ng1894

[21] Hicks S, Wheeler DA, Plon SE, Kimmel M (2011) Prediction of missense mutation functionality depends on both the algorithm and sequence alignment employed. Human Mutation 32: 661–668. doi: 10.1002/humu.21490 21480434 10.1002/humu.21490 PMC4154965

[22] Sabel JL, d'Alencon C, O'Brien EK, Van Otterloo E, Lutz K, et al (2009) Maternal Interferon Regulatory Factor 6 is required for the differentiation of primary superficial epithelia in Danio and Xenopus embryos. Dev Biol 325: 249–262. doi: 10.1016/j.ydbio.2008.10.031 19013452 10.1016/j.ydbio.2008.10.031 PMC2706144

[23] de la Garza G, Schleiffarth JR, Dunnwald M, Mankad A, Weirather JL, et al (2013) Interferon regulatory factor 6 promotes differentiation of the periderm by activating expression of Grainyhead-like 3. J Invest Dermatol 133: 68–77. doi: 10.1038/jid.2012.269 22931925 10.1038/jid.2012.269 PMC3541433

[24] Peyrard-Janvid M, Leslie EJ, Kousa YA, Smith TL, Dunnwald M, et al (2014) Dominant mutations in GRHL3 cause Van der Woude Syndrome and disrupt oral periderm development. Am J Hum Genet 94: 23–32. doi: 10.1016/j.ajhg.2013.11.009 24360809 10.1016/j.ajhg.2013.11.009 PMC3882735

[25] Liu H, Leslie EJ, Jia Z, Smith T, Eshete M, et al (2016) Irf6 directly regulates Klf17 in zebrafish periderm and Klf4 in murine oral epithelium, and dominant-negative KLF4 variants are present in patients with cleft lip and palate. Hum Mol Genet 25: 766–776. doi: 10.1093/hmg/ddv614 26692521 10.1093/hmg/ddv614 PMC4743694

[26] Knight RD, Javidan Y, Zhang T, Nelson S, Schilling TF (2005) AP2-dependent signals from the ectoderm regulate craniofacial development in the zebrafish embryo. Development 132: 3127–3138. doi: 10.1242/dev.01879 15944192 10.1242/dev.01879

[27] Rahimov F, Marazita ML, Visel A, Cooper ME, Hitchler MJ, et al (2008) Disruption of an AP-2alpha binding site in an IRF6 enhancer is associated with cleft lip. Nat Genet 40: 1341–1347. doi: 10.1038/ng.242 18836445 10.1038/ng.242 PMC2691688

[28] Adzhubei IA, Schmidt S, Peshkin L, Ramensky VE, Gerasimova A, et al (2010) A method and server for predicting damaging missense mutations. Nat Methods 7. 10.1038/nmeth0410-248 PMC2855889 20354512

[29] Ng PC, Henikoff S (2003) SIFT: Predicting amino acid changes that affect protein function. Nucleic Acids Res 31. 10.1093/nar/gkg509 PMC168916 12824425

[30] Richards S, Aziz N, Bale S, Bick D, Das S, et al (2015) Standards and guidelines for the interpretation of sequence variants: a joint consensus recommendation of the American College of Medical Genetics and Genomics and the Association for Molecular Pathology. Genet Med 17: 405–424. doi: 10.1038/gim.2015.30 25741868 10.1038/gim.2015.30 PMC4544753

[31] Rost B, Radivojac P, Bromberg Y (2016) Protein function in precision medicine: deep understanding with machine learning. FEBS Letters 590: 2327–2341. doi: 10.1002/1873-3468.12307 27423136 10.1002/1873-3468.12307 PMC5937700

[32] Botti E, Spallone G, Moretti F, Marinari B, Pinetti V, et al (2011) Developmental factor IRF6 exhibits tumor suppressor activity in squamous cell carcinomas. Proceedings of the National Academy of Sciences 108: 13710–13715. 10.1073/pnas.1110931108 PMC3158164 21807998

[33] Moretti F, Marinari B, Lo Iacono N, Botti E, Giunta A, et al A regulatory feedback loop involving p63 and IRF6 links the pathogenesis of 2 genetically different human ectodermal dysplasias. The Journal of Clinical Investigation 120: 1570–1577. doi: 10.1172/JCI40267 20424325 10.1172/JCI40267 PMC2860936

[34] Dougherty M, Kamel G, Grimaldi M, Gfrerer L, Shubinets V, et al (2013) Distinct requirements for wnt9a and irf6 in extension and integration mechanisms during zebrafish palate morphogenesis. Development 140: 76–81. doi: 10.1242/dev.080473 23154410 10.1242/dev.080473 PMC6514306

[35] Tan Ene-Choo, PL EC, Yap Shiao-Hui, Lee Seng-Teik, Cheng Joanne, Pop Yong-Chen, Yeow Vincent (2008) Identification of IRF6 gene variants in three families with Van der Woude syndrome. International Journal of Molecular Medicine 21: 747–751. 18506368

[36] Westerfield M (1995) The zebrafish book: a guide for the laboratory use of zebrafish (Brachydanio rerio): University of Oregon press.

[37] Kimmel CB, Ballard WW, Kimmel SR, Ullmann B, Schilling TF (1995) Stages of Embryonic Development of the Zebrafish. Dev Dynamics 203. 10.1002/aja.1002030302 8589427

[38] Hwang WY, Fu Y, Reyon D, Maeder ML, Tsai SQ, et al (2013) Efficient genome editing in zebrafish using a CRISPR-Cas system. Nat Biotech 31: 227–229. 10.1038/nbt.2501 PMC3686313 23360964

[39] Jao L-E, Wente SR, Chen W (2013) Efficient multiplex biallelic zebrafish genome editing using a CRISPR nuclease system. Proceedings of the National Academy of Sciences 110: 13904–13909. 10.1073/pnas.1308335110 PMC3752207 23918387

[40] Meeker N, Hutchinson S, Ho L, Trede N (2007) Method for isolation of PCR-ready genomic DNA from zebrafish tissues. BioTechniques 43: 610–614. 18072590 10.2144/000112619

[41] Link V, Shevchenko A, Heisenberg C-P (2006) Proteomics of early zebrafish embryos. BMC Developmental Biology 6: 1–1. doi: 10.1186/1471-213X-6-1 16412219 10.1186/1471-213X-6-1 PMC1363346

[42] Thisse C, Thisse B (2008) High-resolution in situ hybridization to whole-mount zebrafish embryos. Nat Protocols 3: 59–69. doi: 10.1038/nprot.2007.514 18193022 10.1038/nprot.2007.514

